# 2023 Chinese expert consensus on the impact of COVID-19 on the management of cardiovascular diseases

**DOI:** 10.1097/CP9.0000000000000043

**Published:** 2023-07-20

**Affiliations:** The Second Hospital of Lanzhou University; Renji Hospital Affiliated to Shanghai Jiaotong University School of Medicine; Shanghai Tenth People’s Hospital; Guangdong Provincial People’s Hospital; West China Hospital, Sichuan University; Shanghai Fourth People’s Hospital; Sichuan University West China Hospital; First Medical Center, PLA General Hospital; Yanbian University Affiliated Hospital; Union Hospital Affiliated to Tongji Medical College, Huazhong University of Science and Technology; Tianjin Chest Hospital; Cardiovascular Hospital Affiliated to Xiamen University; University of California, Davis Medical Center; Shawyifu Hospital Affiliated to Zhejiang University School of Medicine; Virginia Mason Franklin Medical Center; Fuwai Huazhong Cardiovascular Hospital; Peking University Third Hospital; Zhongshan Hospital Affiliated to Fudan University; Shanghai Chest Hospital; Xijing Hospital Affiliated to Air Force Medical University; People’s Hospital of Wuhan University; Second Affiliated Hospital of Chongqing Medical University; First Hospital of Peking University; General Hospital of Ningxia Medical University; Second Affiliated Hospital of Zhejiang University School of Medicine; Peking Union Hospital, Chinese Academy of Medical Sciences; First Affiliated Hospital of Nanjing Medical University; First Affiliated Hospital of Guangxi Medical School; Second Affiliated Hospital of Air Force Medical University; Xinhua Hospital Affiliated to Shanghai Jiao Tong University School of Medicine; First Affiliated Hospital of Henan University of Science and Technology; Shanghai Changzheng Hospital; First Affiliated Hospital of Anhui Medical University; Second Affiliated Hospital of Hainan Medical College; Tongji Hospital Affiliated to Tongji University; Tianjin First Central Hospital; Zhongda Hospital of Southeast University; The First Affiliated Hospital of Xinjiang Medical University; The First Affiliated Hospital of Zhejiang University of Traditional Chinese Medicine; Chongqing People’s Hospital; Zhoupu Hospital Affiliated to Shanghai Health Medical University; Washington University in St. Louis; Hotan District People’s Hospital of Xinjiang; Sixth People’s Hospital Affiliated to Shanghai Jiaotong University School of Medicine; Huashan Hospital Affiliated to Fudan University; Chinese Video Journal of Cardiology; Affiliated Hospital of Zunyi Medical University; Wuhan Asia General Hospital; Peking University People’s Hospital; Wuhan University People’s Hospita; Shanghai First People’s Hospital; Ninth People’s Hospital Affiliated to Shanghai Jiao Tong University School of Medicine; St. Louis Mercy Hospital; Second Affiliated Hospital of Nanchang University; Fuwai Hospital, Chinese Academy of Medical Sciences; First Affiliated Hospital of Dalian Medical University; General Hospital of the Southern Theater Command; Shawyifu Hospital Affiliated to Nanjing Medical University; People’s Hospital of Gansu Province; National Children’s Hospital Toledo Branch; Shanghai Tenth People’s Hospital; Beijing Hospital; The 920th Hospital of the PLA Joint Logistics Support Force; Xiangya Hospital of Central South University; The First Affiliated Hospital of Xi’an Jiaotong University; Shandong Provincial Hospital; The Third Xiangya Hospital of Central South University; The First Affiliated Hospital of Bengbu Medical College; Fuwai Hospital, Chinese Academy of Medical Sciences; Xinhua Hospital Affiliated to Shanghai Jiaotong University School of Medicine; Ruijin Hospital Affiliated to Shanghai Jiaotong University School of Medicine; Zhongshan Hospital Affiliated to Fudan University; Peking Union Hospital, Chinese Academy of Medical Sciences; The First Hospital of Lanzhou University; The First Affiliated Hospital of Xinxiang Medical College; Shanghai Changhai Hospital; China-Japan Friendship Hospital; San Franciscan Heart Center; Zhongshan Hospital Affiliated to Fudan University; Second Xiangya Hospital of Central South University; Beijing Anzhen Hospital Affiliated to Capital Medical University, Beijing 100029, China

**Keywords:** COVID-19, Cardiovascular system, Diagnosis, Treatment, Rehabilitation

## Abstract

The primary site of infection in COVID-19 exhibit is the respiratory system, but multiple organ systems could be affected. The virus could directly invade cardiomyocytes. Alternatively, cytokine storm could lead to myocardial injury. More importantly, the management of existing cardiovascular diseases must be re-examined in COVID-19 due to, for example, interaction between antiviral agents and with a wide variety of pharmacological agents. The Branch of Cardiovascular Physicians of Chinese Medical Doctor Association organized a panel of experts in cardiovascular and related fields to discuss this important issue, and formulated the “2023 Chinese Expert Consensus on the Impact of COVID-19 on the Management of Cardiovascular Diseases.” The Consensus was drafted on the basis of systematic review of existing evidence and diagnosis and treatment experience, and covers three major aspects: myocardial injury caused by COVID-10 and COVID-19 vaccine, the impact of COVID-19 on patients with cardiovascular disease, and the impact of COVID-19 on the cardiovascular system of healthy people, and rehabilitation guidance recommendations. The Consensus involves 11 core clinical issues, including incidence, pathogenesis, clinical manifestations, treatment strategies, prognosis, and rehabilitation. It is our hope that this Consensus will provide a practical guidance to cardiologists in the management of cardiovascular diseases in the new era of COVID-19 pandemic.

## INTRODUCTION

The global pandemic of coronavirus disease 2019 (COVID-19) is caused by severe acute respiratory syndrome coronavirus 2 (SARS-CoV-2). The entire population is susceptible to infection. Similar to other RNA viruses, SARS-CoV-2 mutates as it spreads. The initial strain in 2019 mutated into the delta variant in early 2021 and then into the omicron variant in November of the same year. By the end of 2022, the most prevalent omicron subvariant is BA.5.2 and BF.7 in China and the rest of Asia^[1]^, and BQ.1 in Europe. In the United States, the highly contagious and virulent XBB.1.5 strain now accounts for 40% of the cases^[2]^. As of January 1, 2023, more than 660 million confirmed cases and more than 6.69 million deaths have been reported globally^[3]^.

Coronavirus is a group of single-stranded RNA viruses with a propensity for rapid mutation and recombination, and can cause respiratory or intestinal infections in humans and animals^[4]^. Entry of SARS-CoV-2 to mammalian cells is mediated by the binding of the S protein on viral surface to angiotensin-converting enzyme 2 (ACE2), which in turn is highly expressed in the heart^[4]^. Cardiovascular complications are thus prevalent in COVID-19 patients.

The primary site of infection in COVID-19 is the respiratory system. However, many other systems, including cardiovascular system, are also affected. A previous study showed myocardial injury in 12% of the patients hospitalized for COVID-19^[5]^. Pre-existing cardiovascular diseases increase the risk of severe COVID-19, which in turn exacerbates latent or chronic heart diseases and promotes new cardiac complications^[6]^. The impact of COVID-19 on cardiovascular systems mainly involves three groups of people: (1) patients with myocardial injury caused by COVID-19 infection or vaccination; (2) patients with existing cardiovascular diseases; and (3) previously healthy individuals.

**Figure 1.**
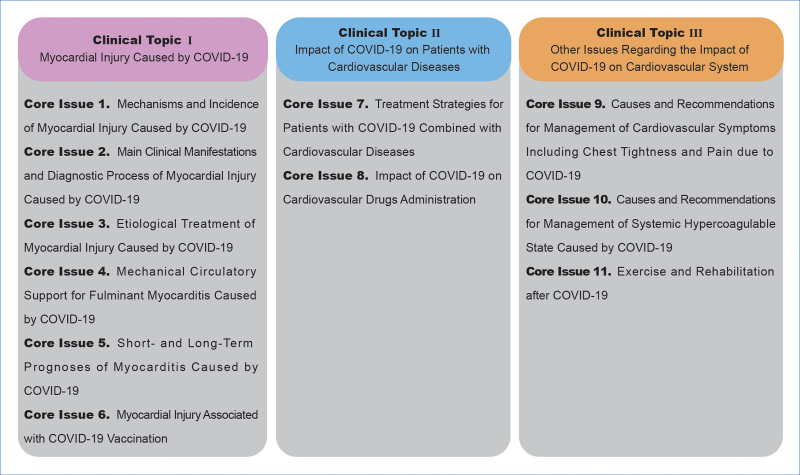
**A Framework of the core elements in the 2023 Chinese Expert Consensus on the Impact of COVID-19 on the Management of Cardiovascular Diseases**. COVID-19: coronavirus disease 2019.

The “2023 Chinese Expert Consensus on the Impact of COVID-19 on the Management of Cardiovascular Diseases” (hereinafter referred to as Consensus) was initiated by the Cardiovascular Physician Branch of the Chinese Medical Association. A panel of experts in relevant fields, both local and abroad, developed the Consensus based on the systematic review of existing evidence and their experience in practice. Consensus opinions were formulated on clinical issues (diagnosis and treatment) of cardiovascular diseases caused or complicated by COVID-19. The Consensus covers three areas and 11 core clinical issues (Figure 1): myocardial injury caused by COVID-19 and vaccine, the impact of COVID-19 on patients with pre-existing cardiovascular diseases, and the other related issues.

## CLINICAL TOPIC I. MYOCARDIAL INJURY CAUSED BY COVID-19

### Core Issue 1. Mechanisms and incidence of myocardial injury caused by COVID-19

#### Direct and indirect mechanisms

In addition to respiratory manifestations, cardiac involvement could be seen in some COVID patients. Putative mechanisms by which SARS-Cov-2 infection causes myocardial injury include: (1) viral invasion of cardiomyocytes through ACE2; (2) excessive inflammatory response and cytokine storm caused by SARS-Cov-2; (3) impact via pathological changes, either systemic or in other organ systems (eg, severe hypoxia).

SARS-Cov-2 can directly infect and damage cardiac cells. Postmortem studies in patients with COVID-19 revealed cardiac hypertrophy, right ventricular dilatation and evidence of right ventricular involvement (19%), lymphocytic myocarditis (14%-40%), focal pericarditis (19%), endocardial thrombosis (14%) or endothelitis, and small vessel thrombosis (19%)^[7–9]^. The high expression of ACE2 in cardiomyocytes renders them susceptible to injury by SARS-Cov-2^[10–12]^. SARS-Cov-2 infection produces cytotoxic effects, activates the innate immunity (eg, interferon signal transduction, apoptosis, reactive oxygen stress [ROS] and antiviral pathways)^[13]^, inhibits metabolic pathways and suppresses ACE2 expression. SARS-Cov-2 particles have been directly seen in EMB tissue with electron microscopy^[10,13]^. Postmortem studies of COVID-19 patients have revealed the presence of RNA of SARS-Cov-2 in myocardial interstitial cells^[14]^. There is a positive correlation between the severity of cardiac involvement and the amount of SARS-Cov-2 in the lungs^[15]^, indicating that myocardial injury caused by SARS-Cov-2 correlates with the severity of pulmonary invasion. A recent study showed that SARS-Cov-2 can cause myocardial fibrosis and myocardial contractile dysfunction via prolonged inhibition of mitochondrial metabolism^[16]^. In a recent study by Nchioua *et al*. in cardiomyocytes differentiated from human induced pluripotent stem cells (iPSC)^[17]^, human cardiomyocytes could be infected by a variety of SARS-Cov-2 subvariants, including the early variant NL-02-2020, delta and omicron (BA.1, BA.2 and BA.5); viral replication generally achieved a maximum in 5 days post-infection. In the study by Nchioua *et al*., viral replication and virulence of BA.5 were higher than BA.1 and similar to the delta strain.

Indirect mechanisms of myocardial injury include hypoxic respiratory failure and hypoxemia, small vessel ischemia and thrombosis, and acute right ventricular failure due to pulmonary embolism or *in situ* pulmonary artery thrombosis. Dysfunctional immune response may also contribute to cardiac injury in COVID-19 patients. Both compromised and hyperactive immunity may worsen COVID-19. Systemic inflammatory responses and cytokine storms can lead to cell death and multi-organ dysfunction. The late autoimmune phenomena are believed to be a consequence of autonomic dysfunction. Key mechanisms of inflammatory and immune dysregulation include hyperinflammatory state, inadequate type I interferon response, adaptive immune dysfunction and antibody-dependent enhancement/impairment^[18,19]^. Mechanisms of vascular thrombosis and platelet activation include indirect activation of platelets and interaction with the innate immune system^[20]^, direct platelet reprogramming^[21]^, and autoimmune phenomena and adaptive immune dysfunction in COVID-19 associated vascular thrombosis^[22]^. Dysfunction of the renin–angiotensin system also contributes to the inflammatory and immune dysregulation^[23]^.

#### Myocarditis caused by COVID-19

Among myocardial injuries caused by COVID-19, myocarditis is relatively uncommon. According to American College of Cardiology (ACC), myocarditis caused by COVID-19 can be classified into three categories: possible, probable, and definitive^[24]^ (Table 1).

**Table 1:** ACC recommendation on the categories of myocarditis by diagnostic certainty

Category	Symptoms	cTn level	ECG/echocar diography	EMB/CMR
Possible myocarditis	Present	Elevated	Abnormal	Normal/not performed
Probable myocarditis	Present	Elevated	Abnormal	Abnormal within 6 months
Definite myocarditis	Present	Elevated	Abnormal	Active myocarditis

ACC: American College of Cardiology; CMR: cardiac magnetic resonance; cTn: cardiac troponin; ECG: electrocardiography; EMB: endomyocardial biopsy.

Myocarditis caused by COVID-19 consists of three phases: acute viral exposure and innate immune response (<1 week); activation of acquired immune response with release of cytokines and chemokines (1–4 weeks); and the development of cardiomyopathy (myocardial fibrosis and remodeling) accompanied along with viral clearance (>4 weeks)^[25,26]^. The duration of myocarditis can be delayed by days to weeks after viral infection.

Precise prevalence of myocarditis caused by COVID-19 is unknown due to a lack of specific diagnostic measures for assessing myocarditis. Many biomarkers that reflect myocardial injury are also associated with non-primary myocardial injury (multi-organ failure, hypoxia, hypoperfusion, and coagulation system activation). In a study during the early COVID-19 epidemic in the United States, it is estimated that in every 1,000 hospitalized patients with COVID-19, there were 2.4 cases of definite or probable myocarditis and 4.1 cases of possible myocarditis^[27]^. The estimated incidence of myocardial injury in patients with severe COVID-19 is 15%–28%. COVID-19 patients with pre-existing heart diseases are at higher risk of myocarditis^[28]^. In COVID-19 patients with acute myocarditis, 39% required positive inotropic support or temporary mechanical circulatory support, and 70.4% were admitted to intensive care unit (ICU)^[27]^.

The incidence of myocarditis following mRNA vaccination of COVID-19 is 56 to 69 cases per 1,000,000 in men and 8 to 10 per 1,000,000 in women^[29]^. In the United States and Israel, the overall incidence rate is 0.3 to 5.0 cases per 100,000 vaccinees^[24]^. The U.S. Food and Drug Administration (FDA) and the European Medicines Agency recently estimated that about 1 in every 100,000 people vaccinated with mRNA vaccines may develop myocarditis, with younger men at higher risk. In Hong Kong, China, the incidence of myocarditis after mRNA vaccination was 0.57 cases per 100,000 vaccinees^[30]^.

### Core Issue 2. Main clinical manifestations and diagnostic process of myocardial injury caused by COVID-19

#### Clinical features

Myocardial injury caused by COVID-19 is common in clinical practice. Diagnosis is based on positive SARS-CoV-2 pathogenicity test plus cardiac troponin I/T (cTnI/cTnT) that exceeds the upper limit of the 99% reference range^[31]^. Clinical manifestations and severity of cardiac injury vary widely among individuals, from mild fatigue and shortness of breath to chest tightness, chest pain, palpitations and syncope, and even cardiogenic shock, malignant arrhythmias, and death.

Myocardial injury caused by COVID-19 in the acute phase mainly manifests as non-specific acute symptoms, including fever, fatigue, cough, sore throat, nasal congestion, runny nose, loss of taste and smell, and diarrhea. These symptoms reflect the activation of the innate immunity. In the second phase of adaptive immune response (typically from the first to fourth weeks), more extensive cardiac involvement may occur, and patients may experience dyspnea, chest tightness, chest pain, palpitations, extreme weakness, and syncope. It is typically in this second phase that patients seek medical help.

Fulminant myocarditis is rare, but significantly increases patient mortality. Accordingly, prompt responses must be made. The symptoms are similar to those of sepsis, and may include pulmonary circulatory congestion (agitation, orthopnea, dyspnea) and cardiogenic shock (hypotension, frailty, pallor, cold and wet skin, cyanosis, spotted skin changes, confusion, irritability or coma, sweating, oliguria, or anuria). Fulminant myocarditis is often accompanied by malignant ventricular arrhythmias.

#### Ancillary examinations

##### Laboratory testing

In patients with a confirmed diagnosis of COVID-19 and precordial discomfort, cTnI/cTnT must be examined as rapidly as possible. Elevated cTnI/cTnT indicates myocardial injury. In contrast to acute myocardial infarction, cTnI/cTnT increase in COVID-19-induced myocarditis is less rapid and lacks a peak. Prolonged elevation of the cTnI/cTnT level suggests the presence of persistent myocardial injury and predicts poor prognosis. Elevated type B natriuretic peptide (BNP) or N-terminal pro-B-type natriuretic peptide (NT-proBNP) is indicative of cardiac insufficiency and is helpful in assessing disease progression, treatment response, and outcomes.

##### Electrocardiography

The most common abnormality in patients with myocarditis caused by COVID-19 is sinus tachycardia, frequent atrial, or ventricular premature beats, followed by supraventricular tachycardia and ventricular tachycardia. Conduction block, manifested as bradycardia, bundle branch block, and atrioventricular block, may also occur. Electrocardiography (ECG) is simple and easy to operate, and has good diagnostic sensitivity in detecting myocarditis but lacks specificity. As a consequence, ECG could be used in monitoring disease progression in patients. Continuous ECG monitoring should be conducted in patients suspected of having arrhythmias but appear normal in conventional ECG.

##### Echocardiography

Patients with mild myocarditis may have normal left heart function. In patients with more extensive myocardial injury, echocardiography may show localized or even global left ventricular systolic dysfunction. Due to the varying degree of injury in different regions, echocardiography may reveal segmental motion abnormalities of the left ventricular wall in non-coronary distribution areas. Patients with fulminant myocarditis often have diffuse ventricular wall motion disturbances, significant decrease in left ventricular ejection fraction, edema and thickening of the interventricular septum and ventricular wall. Since ejection fraction by echocardiography has low sensitivity in detecting abnormality in localized ventricular wall movement and is prone to operator deviation, speckle tracing technique is recommended to evaluate left ventricular systolic function when available.

##### Cardiac magnetic resonance

Cardiac magnetic resonance (CMR) is recommended for non-severe suspected patients with significant precordial discomfort accompanied by elevated cTnI/cTnT but normal ECG and echocardiography to examine the cardiac structure and cardiac function, and to obtain evidence of pathological changes (e.g., myocardial edema, necrosis, and fibrosis). CMR is an important tool to determine whether COVID-19-related myocardial injury is indeed myocarditis. A recent systematic review concluded that two-thirds of patients with myocarditis exhibited varying degrees of late gadolinium enhancement (LGE) in CMR. Diffuse edema due to myocardial inflammation was observed in 19 of 38 patients in the T2-mapping sequence^[32]^. CMR results showing biventricular dysfunction and patchy LGE in the interventricular septum and lower left ventricular wall for more than 3 months are thought to be associated with adverse cardiac events (including sudden death and heart transplantation). Importantly, diffuse edema may be the only CMR change in patients myocarditis associated with COVID-19 due to the absence of clearly recognizable LGE in some patients^[33]^.

##### Endomyocardial biopsy

Endomyocardial biopsy (EMB) is the gold standard for the diagnosis of myocarditis, but not recommended for patients in the acute phase. Inflammatory myocardial infiltration associated with cardiomyocytes degeneration and necrosis has been shown by EMB in patients with myocarditis due to COVID-19. EMB could reveal scattered necrotic cardiomyocytes and CD4^+^ and CD8^+^ lymphocytes near vascular structures in patients with mild cTnI/cTnT elevation, and infiltration of T lymphocytes and CD68^+^ macrophages in patients with more severe COVID-19. No evidence of coronary involvement has been found so far, but endothelial inflammation is common.

#### Diagnostic procedures

**Figure 2.**
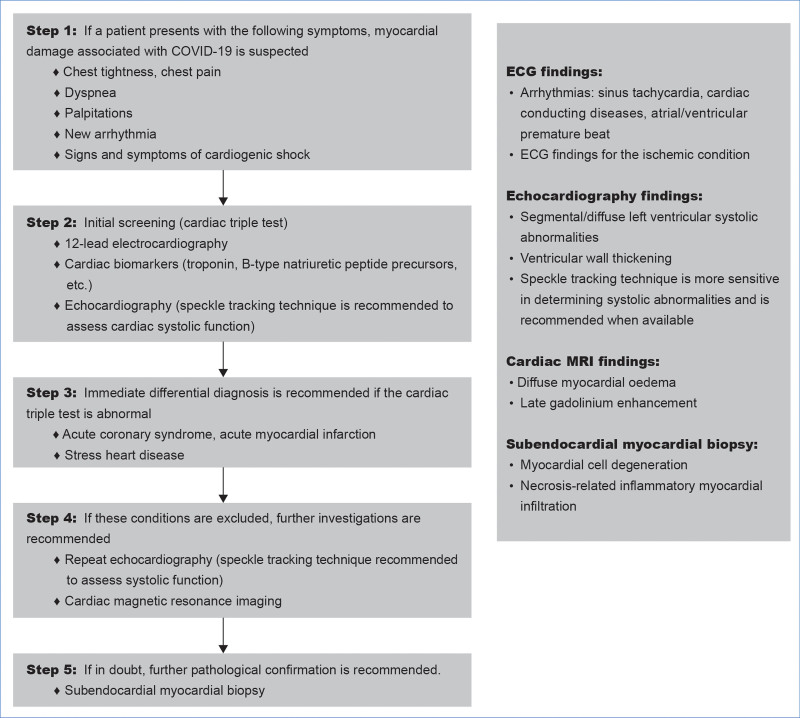
**Flow chart for the diagnosis of myocarditis associated with COVID-19**. COVID-19: coronavirus disease 2019; ECG: electrocardiography; MRI: magnetic resonance imaging.

Based on available information and expert experience in China and abroad^[24]^, we developed a set of standard procedures for the diagnosis and treatment of suspected myocarditis caused by COVID-19, with a goal to maximize patient safety under limited medical resources. Details are available in Figure 2.

### Core Issue 3. Etiological treatment of myocardial injury caused by COVID-19

There have been no large clinical studies on antiviral therapy for COVID-19-associated myocarditis. Since the primary mechanism of COVID-19-associated myocarditis is the immune response and inflammatory storm caused by viral infection, early and effective antiviral therapy could theoretically reduce the risk of myocarditis.

#### Antiviral therapies

##### Paxlovid (nirmatrelvir/ritonavir tablets)

Paxlovid is recommended for adult patients with mild to moderate COVID-19 with high-risk factors for progression to severe disease within 5 days of onset. The recommended dosage is 300 mg nirmatrelvir plus 100 mg ritonavir every 12 h for 5 consecutive days. In the EPIC-HR trial, Paxlovid reduced the risk of progression to severe disease by 89%^[34]^. There has been no direct evidence for reduced risk of myocardial injury. Potential benefit of Paxlovid may interact with the risk level and timing. Drug interaction must be considered. Caution must be exercised in patients with severe hepatic and renal insufficiency (eGFR <30 mL/min/1.73 m^2^) and in pregnant women.

##### Molnupiravir

Molnupiravir is recommended for adult patients with mild to moderate COVID-19 with a high risk for progression to severe disease within 5 days of onset. In the MOVe-OUT trial, molnupiravir reduced the risk of hospitalization and death in COVID-19 patients^[35]^. The recommended dosage is 800 mg twice daily for 5 consecutive days. Molnupiravir is not recommended for use in pregnant and lactating women.

##### Azvudine

Azvudine has been used to treat adult patients with moderate COVID-19, but lacks sufficient evidence^[36]^, particularly on long-term side effects. The recommended dosage is 5 mg/day for no longer than 14 days. Azvudine is not recommended for use in pregnant and lactating women. Caution must be exercised in patients with moderate to severe hepatic or renal impairment.

It must be pointed out that the primary cause of myocardial damage in COVID-19 patients is cytokine storm rather than the virus. Antiviral therapy in COVID-19 patients who have already developed myocarditis is thus highly controversial. In a recent study of patients with severe COVID-19, antiviral therapy with lopinavir-ritonavir and hydroxychloroquine did not reduce the 180-day mortality rate, indeed, there was a trend for increased mortality in patients undergoing antiviral treatment^[37]^. As such, antiviral therapy should be used with extreme caution in patients with severe/fulminant myocarditis.

#### Immunomodulatory therapies

There has been no high-quality evidence for immunosuppressive therapy in myocarditis due to COVID-19. However, considering the similar etiology between myocarditis associated with COVID-19 versus other viral myocarditis and the efficacy of anti-inflammatory therapy in patients with severe COVID-19 in the REMAP-CAP trial^[37]^, we recommend glucocorticoid therapy for patients with myocarditis due to COVID-19 who have developed left ventricular insufficiency or new-onset arrhythmias. There have been no studies on specific glucocorticoid for myocarditis caused by COVID-19. A previous study in hospitalized COVID-19 patients requiring oxygen therapy showed that dexamethasone treatment reduced 28-day mortality^[38]^. We therefore recommend dexamethasone treatment (5–10 mg/d for 5–10 days) or methylprednisolone (40–80 mg/d for 5–10 days). Early treatment with oral prednisone is also recommended. The use of intravenous immunoglobin (IVIG) is controversial. In previous studies of adult patients with respiratory distress syndrome receiving mechanical ventilation, IVIG treatment failed to improve 28-day clinical outcomes. Also, several meta-analyses suggested that IVIG treatment does not reduce the time of hospitalization and mortality in patients with COVID-19^[39,40]^. However, IVIG has been reported to improve the cardiac structural, function and prognosis in patients with fulminant myocarditis^[41,42]^. To summarize, the use of IVIG should be based on comprehensive assessment in individual patients. Efficacy of the interleukin 6 (IL-6) inhibitor tocilizumab has been established in patients with severe COVID-19^[37]^ and therefore may be considered in patients with elevated IL-6. In patients with a high risk of severe disease and high viral load in the early phase of the disease, convalescent plasma (200–500 mL, 4–5 mL/kg) may be considered; reinfusion may be considered depending upon disease progression and viral load.

In conclusion, the benefit/risk ratio of antiviral treatment should be considered based on a comprehensive assessment in individual patients. Paxlovid is the first choice for patients within 5 days of onset. After 5 days, immunomodulatory agents are the mainstay, and antiviral treatment should be used with caution, particularly in patients with fulminant myocarditis.

### Core Issue 4. Mechanical circulatory support for fulminant myocarditis caused by COVID-19

Fulminant myocarditis is characterized primarily by acute onset and rapid progression. Hemodynamic abnormalities and severe arrhythmias could occur rapidly. Fulminant myocarditis is not rare in patients with myocarditis caused by COVID-19. During the early epidemic in the United States, 39% of patients with acute myocarditis presented with fulminant myocarditis and required positive inotropic support or temporary mechanical circulatory support^[27]^.

Once fulminant myocarditis is diagnosed, the disease progresses rapidly. Early mortality is extremely high. Long-term prognosis, however, is relatively benign in patients who survive the early period. The core treatment strategy is early and aggressive treatment, with emphasis on life-support. In addition to general supportive treatment, anti-heart failure therapy and antiviral + glucocorticoid + immunomodulatory therapy, invasive respiratory and circulatory support may be needed^[41]^, as elaborated below.

Fulminant myocarditis due to COVID-19 is reversible. Accordingly, the goal of mechanical circulatory support is to allow sufficient time for cardiomyocytes to recover by reducing the afterload, maintaining systemic and coronary perfusion, and preventing multi-organ dysfunction. Measures for short-term circulatory support include: intra-aortic balloon pump (IABP), extracorporeal membrane oxygenation (ECMO), and left ventricular assist device (Impella)^[43]^.

IABP: Intra-aortic balloon inflates and deflates in synchronization with the cardiac cycle to increase the cardiac output. IABP relies on the rhythmic contraction of the heart. The increase of circulatory flow is limited (10%–20%).

ECMO: ECMO described in this consensus refers to vein-artery ECMO (VA-ECMO). Venous blood is collected via a centrifugal pump, passed through a membrane oxygenator for gas exchange, and then returned to the arteries. VA-ECMO could completely replace the function of the heart and lungs and maintain circulatory function. The arterial return is advective. Left ventricular afterload and myocardial oxygen consumption are therefore increased.

Impella: Impella is a left ventricle-aortic axial-flow assist device that pulls blood out of the left ventricular and pumps it back into the aorta. Impella increases cardiac output, reduces left ventricular work, decreases myocardial oxygen consumption, and increases coronary blood flow.

Each of the three mechanical circulatory support devices has its own advantages and disadvantages. IABP is the most widely used device for mechanical circulatory support due to its simplicity, safety, and effectiveness. ECMO and Impella provide greater flow support and better hemodynamic improvement than IABP, but at higher risk of complications and increased difficulty of management. There is evidence for greater benefit with ECMO than IABP^[44]^. Since VA-ECMO increases the left ventricular afterload, a combination of IABP and ECMO should be considered when satisfactory effects are not achieved using either IABP or ECMO alone^[45]^. Impella could be used in patients with right heart failure to increase right ventricular output and improve right heart function^[46]^. The choice and timing must be based on the hemodynamic condition, as well as the device availability and physician experience.

The use of anticoagulants must be closely monitored since the coagulative function could be affected by COVID-19 per se as well as antiviral agents. Longer-term left ventricular assist device or heart transplant should be considered in patients who require mechanical circulatory support for more than 2–3 weeks^[47]^.

### Core Issue 5. Short- and long-term prognoses of myocarditis caused by COVID-19

#### Prognosis and risk factors

Acute myocarditis may lead to heart failure, often present with reduced ejection fraction, cardiogenic shock, inflammatory shock, and vasodilatory shock^[24,48]^. In a prospective observational study conducted in the United States, definite diagnosis of myocarditis was established in 112 out of 56,963 hospitalized patients with COVID-19, and the overall mortality in these patients was 6.6% within the 120-day follow-up, 15.1% and 0% in patients with and without pneumonia, respectively^[27]^. No data are available on the long-term prognosis of COVID-19^[49]^.

The risk factors of morbid events in patients with COVID-19 myocarditis are generally similar to that in patients with severe COVID-19, more severe complications and higher mortality are often associated with older age and underlying diseases^[50]^, including diabetes, cardiovascular diseases, and respiratory diseases. One study reported that nearly half of the patients with severe COVID-19 had the aforementioned risk factors. Another study reported that nearly 60% of patients with myocarditis caused by COVID-19 had existing underlying conditions, for example, hypertension, diabetes, obesity, asthma, and chronic obstructive pulmonary disease (COPD)^[51]^. Consequently, prognosis in patients with myocarditis caused by COVID-19 is closely related to the underlying conditions of the patients.

As mentioned earlier, pneumonia in patients with acute myocarditis and COVID-19 is a risk of cardiovascular events and poor prognosis^[27]^. The presence of myocardial damage (elevated cardiac enzymes) after COVID-19 usually suggests a poor prognosis. Nevertheless, COVID-19 patients with uncomplicated acute myocarditis have a low risk of serious cardiovascular events, either short- or long-term, after discharge from the hospital^[24]^.

#### Prognosis and exercise

In a meta-analysis of young athletes recovering from COVID-19, the majority did not develop serious cardiac complications. However, sports medicine guidelines recommend that patients who developed acute myocarditis after COVID-19 wait 3 to 6 months before engaging in physical exercise^[52]^. We recommend that in children and adolescents, physical activities after recovery from COVID-19 should be performed under physician supervision^[49,53]^.

### Core Issue 6. Myocardial injury associated with COVID-19 vaccination

#### Clinical benefits of the COVID-19 vaccination

Vaccination against SARS-CoV-2 significantly reduces the risk of infection by all COVID-19 variants (including omicron). A retrospective study of 10.6 million people in the United States showed that with two doses of mRNA vaccination, the risk of infection was reduced by 48% over 27 months; the risk of hospitalization was reduced by 71%–73%; and the risk of death was reduced by 77%–85%^[54]^. The risk of severe COVID-19 after a booster dose was further reduced from 8.8 to 7.6 events/1,000 person-years^[55]^. In patients with no contraindications, additional booster vaccination is recommended^[55]^.

#### Myocarditis caused by COVID-19 vaccination

Existing data suggest COVID-19 vaccination may lead to myocardial injury, myocarditis, and even fulminant myocarditis^[56]^. The estimated incidence of myocarditis associated with COVID-19 vaccination is 56–69 per million in men and 8–10 per million in women^[29]^. Based on the surveillance data from the United States, the incidence of myocarditis in people receiving two doses of mRNA vaccine (including Pfizer BNT162b2 and Moderna mRNA-1273) was 40.6 per million men aged 12–29 years, 4.2 per million women aged 12–29 years, 2.4 per million men aged 30 years and older, and 1.0 per million women aged 30 years and older. The highest incidence of myocarditis was found in males aged 12–17 and 18–24 years, with 62.8 per million and 50.5 per million, respectively. Although 96% of the patients were hospitalized, symptoms were generally mild and there was no report of death^[57]^. In a study from Israel, the incidence of myocarditis in people receiving one dose of mRNA vaccine (Pfizer BNT162b2)^[58]^ was 21 per million in the overall population and 107 per million among men aged 16–29 years^[58]^. In another study from Israel, the incidence of myocarditis was 38.3 per million men and 4.6 per million women in people receiving two doses of mRNA vaccine (Pfizer BNT162b2). The incidence of myocarditis was highest in males aged 16–19 years, 150.7 per million, and in females aged 16–19 years, 10 per million^[59]^. Ninety-five percent of patients with myocarditis had mild symptoms, and there was no reported death due to fulminant myocarditis^[59]^. The incidence of myocarditis varies among mRNA vaccines. A Danish study revealed a higher incidence of myocarditis with Moderna mRNA-1273 vaccine (42 per million) than with the Pfizer BNT162b2 vaccine (14 per million)^[60]^. A higher incidence of myocarditis has also been reported with the Moderna mRNA-1273 vaccine by another retrospective analysis from the United Kingdom^[61]^.

In general, vaccine-associated myocardial injury is very rare, and occurs mostly after the second dose in young men^[56–58]^. In cases of suspected myocarditis, 86% of the patients developed chest pain 2–3 days after vaccination, and some patients had a combination of clinical manifestations such as myalgia, weakness, fever, palpitation, dyspnea, and fatigue^[62]^. CTnI/cTnT typically reached the highest level 3 days after vaccination. BNP and NT-proBNP were slightly elevated in about two-thirds of patients. C-reactive protein was elevated in most patients and decreased when cTnI/cTnT decreased. Common ECG manifestations included ST-segment elevation, diffuse ST-T changes, and ventricular and supraventricular tachycardia, although some patients had no ECG changes. Echocardiographic abnormalities were present in 40% of patients; a small percentage of patients presented with <50% left ventricular ejection fraction. Cardiac MRI images suggest myocarditis, including LGE and myocardial edema^[62]^.

#### Data on myocarditis caused by COVID-19 vaccination from China

Data on vaccination-related myocardial injury/myocarditis in China are scarce. Consistent with foreign studies^[30]^, a retrospective study of 866 patients from Hong Kong reported incidence of myocarditis at 0.31 (95% CI: 0.13–0.66) per 100,000 doses of CoronaVac, a vaccine developed by the Sinovac, and 0.57 (95% CI: 0.36–0.90) per 100,000 doses of Pfizer’s BNT162b2. Adjusted multivariate regression in this study suggested an association between myocarditis with BNT162b2 but not CoronaVac^[30]^. However, the prognosis of patients with myocarditis after the mRNA vaccination was better compared with those without vaccination^[30]^. During the 180-day follow-up period, there was one death (1.0%), one case of dilated cardiomyopathy (1.0%) and two cases of heart failure (1.9%) in 104 patients with myocarditis after BNT162b2 vaccination. In contrast, there were 84 deaths (11.0%), 28 cases of dilated cardiomyopathy (3.7%) and 93 cases of heart failure (12.2%) in patients with virus infection-associated myocarditis. Cox regression suggested 92% reduction in mortality risk due to myocarditis after mRNA vaccination versus those with the viral infection-associated myocarditis^[30]^. There is no data on attenuated live vaccines.

In summary, there are convincing data to support favorable benefit–risk ratio of COVID-19 vaccination in all age groups and in both sexes. For every one million men aged 12–29 years receiving a second dose of the COVID-19 mRNA vaccine, approximately 39–47 cases of myocarditis would be expected, but 560 hospitalisations, 138 intensive care cases, and 6 deaths would be avoided^[24]^. Symptoms were mild, with a good prognosis in the vast majority of mRNA vaccine-induced myocarditis cases^[24]^. We recommend full COVID-19 vaccination for both healthy individuals and patients with stable cardiovascular diseases.

## CLINICAL TOPIC II. IMPACT OF COVID-19 ON PATIENTS WITH CARDIOVASCULAR DISEASES

### Core Issue 7. Treatment strategies for patients with COVID-19 combined with cardiovascular diseases

COVID-19 may have an impact on the development and management of common cardiovascular diseases. Previous studies have indicated that comorbid cardiovascular diseases markedly increase the risk of death in patients with COVID-19. Hence, proper and timely management of both COVID-19 and the existing cardiovascular diseases is critical to improving patient prognosis^[63,64]^. The following are key points for managing common cardiovascular diseases in COVID-19 patients.

#### Acute myocardial infarction

Hypercoagulable state and inflammatory response caused by COVID-19 may increase the incidence of acute myocardial infarction. Limited medical resources due to the COVID-19 pandemic may delay treatment for such patients. We emphasize rapid treatment of acute myocardial infarction regardless of COVID-19 infection. Patients should seek medical attention immediately upon severe chest pain, and the emergency access for acute myocardial infarction treatment in hospitals should be available 24 h^[64]^. Revascularization and subsequent treatments shall be implemented based on the time window recommended by current guidelines. A single operation is recommended for patients with multiple vascular lesions to complete the entire revascularization if possible^[65]^ to minimize the risk of infection^[63]^.

#### Chronic coronary syndrome

The incidence of cardiovascular events is generally low in patients with chronic coronary syndrome. COVID-19 patients with chronic coronary syndrome should first undergo a thorough evaluation, primarily with noninvasive methods to evaluate myocardial ischemia^[66]^, including stress ECG, stress echocardiography, stress myocardial perfusion imaging, and coronary computed tomography angiography (CCTA). Treatment plan must be formulated based on the lab findings as well as symptoms and signs. The majority of patients could be managed with, medical treatment, and revascularization should be postponed until recovery from COVID-19 in patients with low-to-intermediate risk. In patients requiring revascularization due to high risk, the choice of percutaneous coronary intervention (PCI) versus coronary artery bypass grafting (CABG) should be made according to the lesions, with appropriate protective measures. We recommend follow-up via telemedicine if possible to minimize viral transmission. Patients with coronary heart disease and severe COVID-19 are at high risk of gastrointestinal bleeding due to the use of antiplatelet drugs and stress response caused by COVID-19 infection. We thus recommend proton pump inhibitors (PPIs).

#### Heart failure

COVID-19 increases the risk of rehospitalization, mechanical ventilation, and death in patients with heart failure^[67,68]^. Reciprocally, heart failure is a risk of severe COVID-19, thus forming a vicious cycle. Comprehensive evaluation is required to make prudent treatment decisions in such patients. We recommend conducting follow-up via telemedicine in patients with stable chronic heart failure to minimize the risk of infection. Symptoms and signs of acute heart failure overlap with that of COVID-19. We recommend early testing which is sensitive and specific for heart failure, including NT-proBNP, bedside chest X-ray (to check for heart enlargement) and bedside echocardiography. For COVID-19 patients with a diagnosis of acute heart failure episode, treatment is identical to that in patients without COVID-19. It is important to note that COVID-19 patients are prone to develop hypotension for a variety of reasons. It is important to monitor blood pressure and adjust drug doses promptly when using anti-heart failure drugs that can affect blood pressure.

#### Acute pulmonary embolism

COVID-19 patients are at increased risk of thrombosis, most notably, acute pulmonary embolism. Acute pulmonary embolism is a high-risk condition with overlapping symptoms to COVID-19, particularly COVID-19 pneumonia^[69]^. As a consequence, rapid identification of pulmonary embolism is crucial. Pulmonary embolism should be suspected in presents with unexplained hypoxemia, sudden deterioration of respiratory function, new-onset tachycardia, unexplained drop in blood pressure, manifestations of deep vein thrombosis in the lower extremities, and signs of increased right heart load indicated by ECG or echocardiography^[69]^. The diagnostic value of D-dimer for pulmonary embolism tends to be low since COVID-19 alone may also increase D-dimer. CTA of the pulmonary arteries is needed to verify the diagnosis. Once a diagnosis is made, thrombolytic or anticoagulant therapy should be implemented based on guidelines. It is important to note that antiviral drugs approved for COVID-19 could interact with anticoagulants. For details on selecting an anticoagulation regimen, please see Core Issues 8 and 10 of this Consensus.

#### Arrhythmia

Arrhythmia is common in patients with COVID-19^[70]^, particularly in ICU patients. Atrial fibrillation is the most common type, with approximately 15%-20% of hospitalized COVID-19 patients reported to have atrial fibrillation, among which approximately 9.6% are new-onset atrial fibrillation^[71,72]^. This Consensus focuses on the management of atrial fibrillation and provides a brief overview of the use of cardiac electrophysiology.

New-onset atrial fibrillation increases the risk of heart failure, stroke and death in patients with COVID-19. The overall management of atrial fibrillation should follow relevant guidelines. In patients with stable hemodynamics, pharmacological treatment, including rhythm control, ventricular rate control, and anticoagulation, should be considered first. Interactions with antiviral drugs (Core Issue 8 of this Consensus) must be considered when selecting antiarrhythmic and anticoagulant drugs. In patients with unstable hemodynamics, electro-conversion should be conducted, followed by appropriate re-assessment. Catheter ablation of atrial fibrillation should be considered in the following conditions after correcting coagulative abnormality^[67]^: (1) atrial fibrillation leading to tachycardia-induced cardiomyopathy; (2) atrial fibrillation leading to syncope; and (3) atrial fibrillation plus ventricular pre-excitation. After recovery from COVID-19, a comprehensive evaluation should be conducted and treatment should be adjusted if necessary.

Urgent catheter ablation should be considered in patients with ventricular tachycardia/ventricular fibrillation leading to electrical storm if conservative treatment is not effective. Permanent pacemaker implantation should be considered in patients with high-degree atrioventricular block, sick sinus syndrome, and pacemaker battery depletion. Non-urgent cardiac electrophysiologic interventions/procedures should be postponed after recovery from COVID-19 to prevent exacerbation of infection or procedure-related infection.

#### Hypertension

Hypertension is the most common cardiovascular comorbidity in patients with COVID-19, but does not increase the risk of death in COVID-19 patients based on a recent large population-based study^[73]^. Blood pressure fluctuates in hypertensive patients with COVID-19. High fever and diarrhea in the early stage can lead to dehydration, and blood pressure may decrease. The elderly are prone to corresponding postural hypotension. Generalized pain, weakness, and anxiety can increase blood pressure after the fever. As a result, standardized blood pressure measurement at home during this epidemic is important. Treatment of hypertension in COVID-19 patients should conform with the guidelines for the general population with hypertension. In two large randomized controlled trials, neither angiotensin-converting enzyme inhibitors (ACEI) or angiotensin receptor blocker (ARB) increased the risk of adverse events due to COVID-19^[74,75]^. However, it is important to note that Paxlovid interacts with many antihypertensives, especially calcium channel blocker (CCB) (see Core Issue 8 of this Consensus for details). Accordingly, potential interaction must be considered when using Paxlovid. For hypertensive patients with stable blood pressure, routine follow-up using telemedicine is recommended when available to minimize the risk of infection.

#### Congenital heart disease and pulmonary hypertension

Patients with congenital heart disease are at higher risk of heart failure and arrhythmias, but there is no evidence to support increased risk of death in patients with COVID-19 and congenital heart disease. Patients with congenital heart disease plus previous history of diabetes, cyanosis, pulmonary hypertension, renal insufficiency, Eisenmenger syndrome, or hospitalization for heart failure are at additional risk and should be closely monitored, including symptoms, vital signs, and hemodynamics.

Pulmonary hypertension increases the risk of severe disease in COVID-19 patients^[76]^. Reciprocally, COVID-19 may worsen pulmonary hypertension, thus forming a vicious cycle. Consequently, pulmonary artery pressure should be monitored closely, with prompt treatment if necessary. Treatment of pulmonary hypertension generally follows the principles as described in current guidelines.

#### Heart transplantation

Patients with heart transplantation are more vulnerable to developing severe COVID-19 due to immunosuppressive status. Data on these patients are scarce. In several studies with small sizes, symptoms in patients with heart transplantation were similar to that in immunocompetent patients infected with COVID-19, but 81.8% required hospitalization with 24.2% of the hospitalized patients on mechanical ventilation^[77]^. Apparently, these patients are at a higher risk of developing severe disease once they are infected. Treatments for such patients include continuing use of immunosuppressive therapy, high-dose glucocorticoids, immunoglobulins, and tocilizumab^[77]^, but there has been no clinical evidence to support the effectiveness of these treatments. Notably, patients with heart transplantation have a markedly lower chance of being infected due to protective measures^[78]^.

#### Other cardiovascular diseases

Treatment strategies for valvular heart disease and cardiomyopathy may need to be adjusted in individual COVID-19 patients: (1) Elective surgery in patients with stable conditions should be postponed to after recovery from COVID-19; (2) Routine follow-up via telemedicine is encouraged in patients with in stable conditions; (3) Interaction between cardiovascular drugs and antiviral drugs must be carefully considered. Observational studies suggest that COVID-19 may lead to the development of stress cardiomyopathy^[79]^. Appropriate examinations should be conducted for differential diagnosis in patients with chest pain plus elevated myocardial biomarkers and abnormal ECG, echocardiography and left ventriculography.

### Core Issue 8: lmpact of COVID-19 on cardiovascular drugs administration

#### Current treatment with cardiovascular drugs

Intensive management of patients with existing cardiovascular disease is necessary during the COVID-19 epidemic, since these patients are at a higher risk of developing severe COVID-19 once infected. Although COVID-19 may cause chronic cardiovascular instability, interruption of medication for cardiovascular disease can also result in serious adverse consequences. Patients with existing cardiovascular disease must consult cardiovascular specialists before making any medication changes.

##### Antiplatelet drugs

Antiplatelet drugs are the most essential drugs for the secondary prevention in patients with coronary heart disease. In patients with atherosclerotic cardiovascular disease, antiplatelet drugs should be used in strict accordance to the guidelines for cardiovascular diseases^[63]^. In patients with coronary heart disease who become infected, especially in patients undergoing coronary intervention or surgery within 6 months, antiplatelet agents should be continued and bleeding should be closely monitored.

Aspirin can potentially block the progression of COVID-19 through its anti-inflammatory, antiplatelet aggregation, and multiple effects on endothelial function. A meta-analysis suggested a potential therapeutic benefit of aspirin in patients with COVID-19, but there are no consistent findings with regards to dosing across different studies. Reduced risk of mortality has been shown at 80–100 mg/day before or during hospitalization, but not at a higher dose of 150 mg/day^[80]^.

##### Angiotensin-converting enzyme inhibitors/angiotensin receptor blockers

Hypertension, coronary heart disease and heart failure are the most common cardiovascular comorbidities in patients with COVID-19. In addition to controlling blood pressure in hypertensive patients, ACEI/ARB are commonly used in secondary prevention of coronary heart disease and treatment of chronic heart failure. In patients with hypertension, the prognosis improves after treatment with ACEI/ARB agents, regardless of the complication of coronary artery disease, heart failure and diabetic nephropathy. Early in the COVID-19 epidemic, there was a concern that ACEI/ARB may increase the risk of COVID-19 and the rate of severe illness^[81]^. However, the latest meta-analysis suggested that the use of ACEI/ARB agents did not increase patient susceptibility to COVID-19 and the rate of severe illness and mortality^[82]^.

Discontinuation of ACEI/ARB agents during hospitalization in patients with combined heart failure increases the likelihood of cardiac failure and has been associated with higher post-discharge mortality^[83]^. We recommend that patients with cardiovascular disease who are already taking ACEI/ARB agents continue to take these drugs. However, cautions should be exercised. Hypotension may occur in patients with COVID-19 due to dehydration, infectious shock or hemodynamic instability, so blood pressure should be closely monitored and the dosage should be adjusted if necessary^[84]^.

##### Other cardiovascular drugs

Drug interactions, particularly with antiviral agents, are important considerations in the treatment of COVID-19^[84]^. For example, when warfarin is used in combination with dexamethasone or methylprednisolone, the international normalized ratio (INR) should be closely monitored. Caution should be exercised when combining antiviral drugs with antiarrhythmic drugs that prolong the QT interval or low-dose digoxin. Apixaban and rivaroxaban should be avoided when oral anticoagulants that are not vitamin K antagonists are needed. For statins, it is recommended to start with low doses of rosuvastatin or atorvastatin. When using colchicine, dosage reduction should be considered for drugs such as statins and CYP3A4 inhibitors (e.g., amiodarone, verapamil, and diltiazem)^[84]^. Close ECG monitoring should be conducted when combining chloroquine or hydroxychloroquine with beta-blockers or drugs that prolong the QT interval.

Progression of COVID-19 is often accompanied by coagulation disorders, and in hospitalized patients with COVID-19, treatment with low-molecular heparin is associated with reduced risk of mortality and thrombosis versus regular heparin, without increased risk of bleeding. We recommend low-molecular heparin as the first choice for anticoagulation during hospitalisation^[85]^.

#### Concurrent use of Paxlovid

Paxlovid is recommended in adult patients with mild to moderate COVID-19 and high-risk factors for progression to severe disease^[38]^. Both nirmatrelvir and ritonavir are substrates of CYP3A. Accordingly, drug that affects the activity of CYP3A metabolizing enzymes affects the metabolism of nirmatrelvir and ritonavir, and ultimately efficacy and safety.

Paxlovid has a variety of drug–drug interactions (DDIs) with commonly used cardiovascular drugs^[86]^. A full list of the DDIs is available at https://www.covid19-druginteractions.org/checker. The DDIs between Paxlovid and commonly used cardiovascular drugs are summarized in Table 2.

**Table 2:** Drug–drug interactions between COVID-19-related drugs and commonly used cardiovascular drugs

Cardiovascular drugs	Effect of paxlovid/azvudine on plasma concentration
Antiplatelet drugs	
Aspirin/Indobufen	No effect
Clopidogrel	Paxlovid reduces plasma concentration and increases thrombotic risk
Prasugrel	Paxlovid reduces plasma concentration without affecting clinical efficacy
Ticagrelor	Paxlovid elevates plasma concentration, and raises risk of bleeding (contraindicated)
Anticoagulants	
Apixaban/edoxaban/dabigatran etexilate	Paxlovid elevates plasma concentration and increases the risk of bleeding; Azvudine reduces the plasma concentration and increases the risk of thrombotic risk
Rivaroxaban	Paxlovid elevates plasma concentration, and increases risk of bleeding (contraindicated)
Warfarin	Paxlovid increases or decreases the concentration of warfarin in different conformations, the risk of bleeding and thrombosis may increase, and INR needs to be monitored
Antilipemic agents	
Atorvastatin/rosuvastatin	Paxlovid increases plasma concentration, increases muscle damage and liver toxicity; Atorvastatin may reduce the concentration of Azvudine, and weaken the antiviral effect
Simvastatin/lovastatin	Paxlovid increases plasma concentration and raises muscle damage and hepatotoxicity (contraindicated)
Ezetimibe	Paxlovid reduces plasma concentration and diminishes clinical efficacy
Pravastatin/fluvastatin/pitavastatin	No effect
Evolocumab/alirocumab	No effect
Antianginal drugs	
Metoprolol/carvedilol/propranolol	No effect
Labetalol/nitrendipine and atenolol	Paxlovid decreases plasma concentration and diminishes the hypotensive effect
Ranolazine	Both antivirals increase plasma concentration and prolongs QT interval (contraindicated)
Anti-heart failure drugs	
Digoxin	Paxlovid may increase plasma concentrations, increase risk of cardiac conducting diseases and nausea and vomiting
Lisinopril/enalapril/captopril	No effect
Candesartan/telmisartan/olmesartan	No effect
Irbesartan	Paxlovid decreases plasma concentration and diminishes the antihypertensive effect
Sacubitril valsartan/losartan/valsartan	Paxlovid elevates plasma concentration and increases antihypertensive effects
Spironolactone	No effect
Eplerenone	Paxlovid raises plasma concentration and increases risk of hypokalemia (contraindicated)
Canagliflozin	Paxlovid reduces plasma concentration and diminishes the hypoglycemic effect
Empagliflozin	No effect
Dapagliflozin	No effect
Others antihypertensive drugs	
Diltiazem/verapamil	Paxlovid raises plasma concentration and increases the risk of hypotension, edema, bradycardia and vertigo
Terazosin	Paxlovid elevates plasma concentration and increases the risk of hypotension
Amlodipine/Nifedipine/Felodipine	Paxlovid increases plasma concentration, increases the risk of hypotension, edema, and facial flushing; those CCBs may increase the concentration of Azvudine, and may increase transaminase levels
Hydrochlorothiazide	No effect
Antiarrhythmic drugs	
Amiodarone/dronedarone/propafenone/quinidine	Paxlovid increases plasma concentration and elevates the risk of malignant arrhythmias (contraindicated); amiodarone/dronedarone may increase azvudine concentrations, may increase transaminase levels
Sotalol	No effect
Drugs to reduce pulmonary hypertension	
Bosentan	Paxlovid elevates plasma concentration and increases risk of headache and vomiting liver injury (contraindicated)
Sildenafil	Paxlovid increases plasma concentration and adds risk of sustained erection with hypotension (contraindicated)
Iloprost/treprostinil	No effect
Immunosuppressive agents	
Cyclosporine/tacrolimus/rapamycin	Paxlovid elevates plasma concentration and increases drug toxicity (contraindicated); those agents may increase Azvudine concentrations, may increase transaminase levels
Anti-inflammatory drugs	
Colchicine	Paxlovid raises plasma concentration and increases drug toxicity (contraindicated)
Dexamethasone/methylprednisolone/prednisone	Paxlovid elevates plasma concentration and raises side effects such as drug-related Cushing’s syndrome
Prednisolone	No effect

CCB: calcium channel blocker; INR: international normalized ratio; QT interval: the time from the start of the Q wave to the end of the T wave.

##### Antiarrhythmic drugs

Paxlovid increases the plasma concentration of many antiarrhythmic drugs. Theoretically, it is relatively safe and reasonable to start Paxlovid 2–2.5 days after discontinuation of antiarrhythmic drugs. This may be challenging in clinical setting. We recommend considering alternative antiviral regimens. The antiarrhythmic agent sotalol is cleared by the kidneys, and that does not interact with Paxlovid, thus, these two can be used together.

##### Antiplatelet drugs and anticoagulants

Antiplatelet drugs are used to treat coronary artery disease, particularly in patients undergoing PCI. It is safe to combine aspirin and prasugrel with Paxlovid. When used in combination with clopidogrel, Paxlovid increases the risk of thrombosis. When used in combination with ticagrelor, Paxlovid increases the risk of bleeding. If possible, clopidogrel and ticagrelor should be replaced with prasugrel. In patients with contraindication to prasugrel, an alternative antiviral regimen should be considered.

Anticoagulants, such as warfarin, can be given in combination with Paxlovid, but close monitoring of blood coagulation markers is required. Paxlovid increases plasma levels of all direct oral anticoagulants. Dose adjustment, temporary discontinuation, and use of alternative agents may be required.

##### Statins

Paxlovid increases plasma levels of simvastatin and lovastatin, and may trigger muscle damage and rhabdomyolysis. Therefore, both drugs must be stopped before starting Paxlovid. Atorvastatin and rosuvastatin could be used in combination with Paxlovid, but require dose reduction. Other statins are generally safe when used in combination with Paxlovid.

Azvudine is an oral small-molecule inhibitor of the human immunodeficiency virus reverse transcriptase, and has been approved for use in adult patients with moderate COVID-19^[36]^. Azvudine increases the plasma level of P-glycoprotein (P-gp) substrates, including empagliflozin, digoxin, ranolazine, edoxaban and dabigatran etexilate, through DDI. The plasma level of azvudine increases when used in combination with P-gp inhibitors, for example, antifungals, amiodarone, and verapamil^[87]^.

In conclusion, patients with cardiovascular disease should continue to take their prescribed cardiovascular medications during the COVID-19 epidemic. Patients with cardiovascular disease who become infected should consult with a cardiovascular specialist before antiviral treatment to adjust their medications^[84]^.

## CLINICAL TOPIC III. OTHER ISSUES REGARDING THE IMPACT OF COVID-19 ON CARDIOVASCULAR SYSTEM

### Core Issue 9. Causes and recommendations for management of cardiovascular symptoms including chest tightness and pain due to COVID-19

Chronic COVID-19 syndrome, also known as post-acute sequelae of COVID-19 (PASC), refers to a range of symptoms that persist during and after recovery from COVID-19. Chronic COVID-19 syndrome typically lasts for 4 to 12 weeks, but could persist for a longer period of time^[24]^.

PASC patients can be categorized into two groups: those with identifiable cardiovascular disease (PASC cardiovascular disease, PASC-CVD) and those with normal lab results or those with symptoms that cannot be fully explained by the lab results (PASC cardiovascular syndrome, PASC-CVS). This Consensus focuses on PASC-CVS and proposes a patient-centered approach to assessment and management^[24]^.

#### Causes of cardiovascular system symptoms caused by COVID-19

The initial presentation of PASC may vary from asymptomatic infection to the emergence of critical illness. Symptoms of the cardiovascular system include palpitation, chest pain, dyspnea upon activity, and exercise intolerance^[88]^. Patients with mild COVID-19 and no underlying cardiovascular disease or previous history may experience PASC symptoms during the recovery period of COVID-19, leading to a decline in health status and quality of life^[89]^.

Mechanisms of PASC-CVS include inflammation, immune activation, persistence of the virus, triggering of latent virus, endothelial dysfunction, motor and metabolic impairment, and severely reduced cardiac adaptability following viral infection. These mechanisms may operate in a single patient, but inflammation and immune activation are the most important. SARS-CoV-2 can enter the local immune system of cardiac and vascular tissues via macrophage CD209 receptors and initiate a cytokine release syndrome in response to the increase and stimulation of multiple cytokines. Loss of CD8^+^ T-cell function following persistent infection can further exacerbate the antiviral immune imbalance, leading to endothelial cell dysfunction and impaired mitochondrial and energy metabolism in cardiomyocytes, which in turn trigger PASC-CVS-related symptoms^[84]^.

#### Management of cardiovascular system symptoms induced by COVID-19

**Figure 3.**
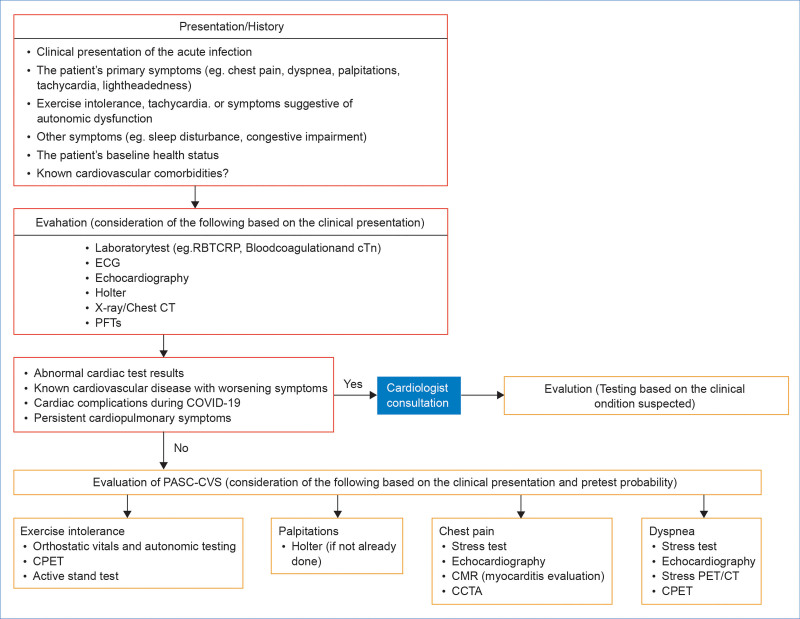
**Clinical pathway for evaluation of PASC-CVD/PASC-CVS**. CCTA: coronary computed tomography angiography; CMR: cardiac magnetic resonance; COVID-19: coronavirus disease 2019; CPET: cardiopulmonary exercise test; CRP: C-reactive protein; CT: computed tomography; cTn: cardiac troponin; ECG: electrocardiography; PASC-CVD: post-acute sequelae of COVID-19-cardiovascular disease; PASC-CVS: post-acute sequelae of COVID-19-cardiovascular syndrome; PET: positron emission tomography; RBT: regular blood test.

In COVID-19 patients who develop symptoms suggestive of PASC (e.g., palpitation, chest tightness, chest pain, and dyspnea after activity) during the infection or recovery period, community physicians, emergency physicians, and general practitioners should assess clinical presentation and history, and perform basic laboratory tests according to the recommended clinical pathway for PASC-CVD/PASC-CVS. Cardiovascular specialists should be consulted if symptoms persist or worsen, existing cardiovascular disease deteriorates, or upon abnormal cardiac findings or definite cardiac complications. Cardiovascular specialists should first verify or exclude myocarditis and PASC-CVD based on medical history and symptoms, as shown in detail in Figure 3.

Empirical treatments may be considered in patients with chest pain without evidence of ischemia. If the chest pain is caused by pleurisy or local inflammation (e.g., costochondritis), a 1- to 2-week diagnostic treatment with non-steroidal anti-inflammatory drugs (NSAIDs) may be considered, low-dose colchicine may be added if necessary. If symptoms worsen despite the use of NSAIDs, esophagitis, and esophageal spasm should be considered. In patients with suspected endothelial dysfunction, treatment with calcium channel blockers, long-acting nitrates, and/or ranolazine may be attempted. Nutritional supplements (e.g., beetroot extract and L-arginine) that increase the generation of nitric oxide can be used in patients with chest pain or microcirculatory disorders that do not respond to other therapies. A recent study suggested that nicotinamide adenine dinucleotide (NAD) could improve metabolism and promote recovery in patients with PASC-CVS^[90]^. In a recent trial, the herbal medication formula Qi Dong Yi Xin improved enzymatic spectrum in patients with myocarditis, indicating a potential as a treatment for myocardial injury in COVID-19 patients^[91]^. In patients with dyspnea that are not attributable to cardiovascular disease, respiratory specialists should be consulted.

Several observational and interventional clinical studies on PASC are currently ongoing. The National Institutes of Health (NIH)-funded RECOVER study focuses on patient mortality within 30 days after discharge of COVID-19 patients in an attempt to clarify the epidemiological features and pathology of PASC. The CORFU study evaluates changes in cardiac function in PASC patients during hospitalization and after discharge by serological tests, echocardiograph, ECG and CMR to clarify the impact of PASC-CVD/PASC-CVS on quality of life and long-term adverse events.

To sum up, the effect of PASC-CVD and PASC-CVS on cardiovascular symptoms in patients with COVID-19 varies from person to person. Due to the risk of myocarditis, PASC-CVD and PASC-CVS, the burden on the cardiovascular system should be minimized during the treatment of COVID-19. We recommend rest for sufficient duration until complete recovery^[56]^.

### Core Issue 10. Causes and recommendations for management of systemic hypercoagulable state caused by COVID-19

About 20% of patients with COVID-19 experience coagulopathy, and almost all patients with severe and critical cases have distinct coagulation disorders^[92]^. The ACTV-4B trial reported elevated D-dimer in approximately 10% of all COVID-19 patients^[93]^. The hypercoagulable state caused by COVID-19 can increase the rates of severe illness and mortality^[93]^. COVID-19 associated coagulopathy can manifest as microthrombi and macrothrombi, leading to damage to multiple organs, including the lungs, heart, brain and kidneys^[94]^. Venous thromboembolism (VTE) in coagulopathy is particularly common in patients with COVID-19. In studies from France, the Netherlands, the United Kingdom, and the United States, the estimated incidence of VTE ranged from 8% to 69%^[95]^. The incidence of pulmonary embolism in patients with severe COVID-19 is twice as high as that observed in patients with influenza, and 90% of patients had no evidence of deep vein thrombosis, indicating that pulmonary embolism is newly formed in most cases^[96]^. Coagulopathy may also trigger other embolic events, including ischemic stroke^[97]^, ST-segment elevation myocardial infarction, and limb ischemia^[98]^.

The cellular and molecular mechanisms by which coagulopathy occurs in COVID-19 patients are not clear, but may involve complex interactions between innate immune responses, coagulation and fibrinolytic pathways, and the vascular endothelium^[94]^. Three major pathological mechanisms have been proposed: vascular endothelial dysfunction, hyperinflammatory immune response, and hypercoagulability^[94]^. Factor VIII activity, fibrinogen concentration, plasminogen activator inhibitor-1 (PAI-1), von Willebrand factor (VWF), tissue factor expression and thrombin production were significantly elevated and platelet activation was also increased in COVID-19, whereas antithrombin, protein C and thrombomodulin levels were reduced, resulting in a hypercoagulable state and thrombosis in some patients^[95]^.

Most VTE events are preventable. A study of 2773 patients hospitalized with COVID-19 by Paranjpe *et al*.^[99]^ showed prophylaxis with anticoagulants decreased VTE from 62.7% to 29.1% and increased the median survival from 9 to 21 days. The benefit was significant even after adjusting mechanical ventilation. They also identified an association between longer duration of anticoagulant use with lower risk of in-hospital death (14% reduction with each additional day of anticoagulant use).

The Expert Consensus of VTE Prevention and Treatment developed by a panel of multidisciplinary experts from Europe and China emphasizes the risk of VTE in all patients with severe or critical COVID-19, and recommends VTE prevention and treatment if there are no contraindications^[92]^, including: (1) Monitoring of coagulation. All patients hospitalized with COVID-19 should be dynamically monitored for changes in platelet count, prothrombin time (PT), activated partial thromboplastin time (APTT), fibrinogen, INR, and D-dimer. (2) Anticoagulation therapy. Low-molecular-weight heparin or unfractionated heparin are recommended in the absence of contraindications in patients with severe or critical COVID-19. In patients with mild or moderate COVID-19, IMPROVE score should be used to assess the risk of embolism. In patients with moderate to high-risk COVID-19, low-molecular-weight heparin or unfractionated heparin should be used if there is no contraindications. In COVID-19 patients with elevated D-dimer, low-molecular-weight heparin is strongly recommended; unfractionated heparin could also be used with close monitoring of the coagulative function. In patients with severe and critical COVID-19 and VTE, the treatment should conform to the current guidelines for VTE: low-molecular-weight heparin should be given at therapeutic dosage; unfractionated heparin could also be used with monitoring of coagulative function. In patients with clear contraindications to heparin use, oral anticoagulants (eg, rivaroxaban) are recommended. We recommend thromboprophylaxis and related measures in all patients hospitalized with COVID-19.

In summary, COVID-19 and the associated hyperinflammatory immune response result in dysfunction of the coagulation/fibrinolytic system and increase the risk of thromboembolism through a variety of mechanisms. Prophylactic anticoagulation is thus required, particularly in patients with a severe or critical disease.

### Core Issue 11. Exercise and rehabilitation after COVID-19

Myocarditis is a common cause of sudden cardiac death in athletes. There are limited data with regards to early return to play (RTP) after recovery from COVID-19. Accumulating studies showed that myocarditis due to COVID-19 in professional athletes accounts for 0.6%–2.3% of the general patient population^[100–102]^. The following is a list of expert recommendations for professional athletes, collegiate athletes, and high-level amateurs.

#### Return to play

Previous RTP guidelines recommend self-quarantine and abstinence from exercise for 10 days after the onset of COVID-19 since symptoms may worsen^[53,103]^. Current evidence, however, demonstrated a low incidence of myocarditis in patients with mild COVID-19. Accordingly, there is no need for strict exercise control. In patients with suspected myocarditis or myocardial involvement, a triad test consisting of 12-lead ECG, high-sensitivity troponin, and echocardiograph is recommended. CMR is needed to verify or rule out myocarditis in the event of abnormality in any of the three tests or normal testing but persistent cardiopulmonary symptoms (chest pain/chest tightness, palpitation, dyspnea, dizziness/syncope, and/or hospitalization due to suspected cardiac involvement).

In 2020, the *British Journal of Sports Medicine* published recommendations on the return to training after COVID-19 for professional athletes^[104]^. In athletes with no COVID-19 symptoms and signs, no additional cardiac function tests are required prior to RTP. In athletes with mild to moderate symptoms of COVID-19 but have fully recovered without persistent cardiopulmonary symptoms, a rest period of 7 days and no earlier than day 10 after the onset of symptoms is recommended, with additional cardiac testing, including 12-lead ECG and echocardiograph, prior to starting RTP. In athletes with persistent symptoms of COVID-19, at least 14 days of rest are required before considering return to training. In athletes with COVID-19 symptoms that require hospitalization, a comprehensive cardiac test panel, including 12-lead ECG, CMR, cardiopulmonary exercise testing and 24-h ambulatory ECG, is recommended^[104]^. The 2022 ACC recommends asymptomatic athletes stop exercising for 3 days after testing positive. Athletes with mild to moderate COVID-19 should wait until symptoms disappear before resuming exercise. Athletes with cardiopulmonary symptoms should complete a comprehensive assessment after resting for at least 5 days and until symptoms disappear before resuming training^[24]^.

Salman *et al*. formulated a practical guideline for returning to physical activity after COVID-19 in non-athletes^[105]^. The guideline recommends risk stratification for people with COVID-19 before they return to physical activity. Further clinical assessment is required for patients with persistent symptoms or those with severe symptoms or a history of cardiac involvement. Exercise should only be resumed after at least 7 days of symptom remission and at a minimum intensity for at least 14 days. Daily self-monitoring is recommended to track the recovery and knowing when to seek professional medical help is mandatory^[105]^. This guideline divides exercise recovery into five phases: preparation for return to training, low-intensity training, moderate-intensity aerobic and strength training, moderate-intensity aerobic and strength training plus coordination and skill training, and full return to the previous level^[105]^. The guideline recommends at least 7 days for each phase and return to the previous level if unable to complete a phase and moving on to the next phase until significant improvement^[105]^.

To sum up, this Consensus recommends 3 days of rest before resuming stepwise training in asymptomatic COVID-19 patients and 7 days of rest starting from symptom disappearance before resuming stepwise training in patients with mild to moderate COVID-19. For COVID-19 patients with severe cardiopulmonary symptoms, training could resume after 14 days after symptom disappearance in those with normal triad test (12-lead ECG, high-sensitivity troponin, and echocardiograph). CMR should be conducted in patients with abnormal triad test to rule out myocarditis. A repeat cardiac triad test is necessary upon re-emergence or worsening of cardiopulmonary symptoms during the return to exercise.

#### Myocarditis and exercise training restrictions

Relevant guidelines recommend exercise restriction for 3–6 months in patients with a diagnosis of myocarditis^[106]^. RTP should be based on: (1) the absence of cardiopulmonary symptoms; (2) recovery of laboratory test indicative of myocardial injury; (3) normal left ventricular systolic function; and (4) absence of spontaneous or induced arrhythmias on the ECG under a challenge test. In the Big Ten COVID-19 registry study, 37% of the athlete participants developed CMR-confirmed myocarditis 4 to 14 weeks after the first positive SARS-CoV-2 test, 11 of these athletes recovered with a median recovery time of 8 weeks, one had a longer recovery time of 10 weeks^[102]^. In conclusion, athletes and non-athletes with myocarditis following definite COVID-19 diagnosis should stop training for at least 3 to 6 months and undergo a rigorous assessment prior to resuming stepwise recovery training.

#### Recommendation for post-acute sequelae of COVID-19

PASC can be divided into short-term and long-term, with no clear boundary. A recently published meta-analysis suggested that 45% of people recovering from COVID-19 still had at least one persistent symptom (e.g., fatigue, sleep disorder, pain/discomfort, dyspnea) after 4 months^[107]^. Regardless of the category (short-term vs. long-term), patients must be assessed to determine the presence of absence of cardiopulmonary symptoms. If cardiopulmonary symptoms are still present, RTP should be initiated based on the above-mentioned protocol. If cardiopulmonary symptoms are no longer present, cardiopulmonary exercise tests and pulmonary function tests should be conducted to devise individualized stepwise recovery training program.

## CONCLUSIONS

In conclusion, COVID-19 can cause myocardial injury, and in some cases, myocarditis or fulminant myocarditis through direct or indirect effects. Cardiologists need to systematically assess patients based on evidence-based medicine as well as empirical findings and develop appropriate treatment plans based on risk stratification. In the new phase of controlling the COVID-19 pandemic, we will face a wide range of issues, including treatment planning and medication adjustment, management of cardiovascular symptoms and resuming exercise after recovery in patients with existing cardiovascular disease as well as cardiovascular symptoms that emerge after infection. This consensus summarizes the latest evidence and clinical experience on the above issues, and provides a list of pragmatic advises to cardiovascular specialists on the management of cardiovascular diseases during the COVID-19 pandemic.

## TAKING-HOME MESSAGES

1. COVID-19 can cause myocardial damage through both direct (viral attack on cardiomyocytes) and indirect action (systemic inflammatory response, cytokine storm and hypoxic state damaging myocardial tissue triggered by the virus). Data from the United States in early 2020 showed that the incidence of confirmed and probable myocarditis was 2.4‰ and 4.1‰ for the suspected myocarditis in hospitalized patients with COVID-19. Myocardial damage was present in 14%–28% of the patients with severe COVID-19.
2. Major assessment tools for myocardial damage due to COVID-19 include: signs and symptoms, cardiac triad test (ECG, echocardiogram, and myocardial enzyme profile). Definitive diagnosis of myocarditis requires CMR or subendocardial biopsy.
3. Etiological treatment of myocardial damage due to COVID-19 consists of antiviral therapy with Paxlovid or azvudine during early infection, glucocorticoid and immunomodulatory therapy in patients with severe disease, and mechanical circulatory support (e.g., IABP, ECMO, and Impella) in patients with fulminant myocarditis.
4. Myocardial damage due to COVID-19 has a favorable prognosis, but the estimated mortality rate in COVID-19 patients with acute myocarditis is 6.6%.
5. Vaccination significantly reduces the rate of severe illness, hospitalization, and mortality after COVID-19. Myocardial injury due to COVID-19 vaccination is rare. The estimated incidence of myocarditis caused by vaccination is 3 to 69 cases per 1 million doses. The benefits of vaccination outweigh the risks. We recommend vaccination in all healthy individuals and patients with stable cardiovascular disease.
6. Patients with existing cardiovascular disease should, in general, maintain ongoing treatments after COVID-19. We particularly emphasize that patients with acute severe cardiovascular conditions (e.g., acute myocardial infarction and pulmonary embolism) should seek medical help as early as possible. Hospitals should make all efforts to maintain chest pain centers open 24 h.
7. In general, existing cardiovascular disease can be treated as usual in COVID-19 patients. However, antiviral drugs (e.g., Paxlovid) interact with drugs that are metabolized by CYP3A. Most arrhythmic drugs (except sotalol) must be discontinued. Some statins need to be reduced. Aspirin can be used as usual.
8. Rest and observation are recommended in patients with mild cardiovascular symptoms such as chest tightness and palpitation. If symptoms persist or worsen, cardiac triad test (ECG, echocardiogram, and myocardial enzyme profile) is recommended. Upon abnormal test, cardiovascular specialist consultation is recommended and if necessary, cardiac MRI or EMB is considered to verify or rule out myocarditis.
9. COVID-19 may trigger a hypercoagulable state. Patients with definite symptoms of chest pain are advised to seek prompt medical help for possible arterial embolic disease (e.g., acute myocardial infarction and pulmonary embolism). D-dimer should be dynamically monitored in bedridden or hospitalized patients; anticoagulative therapy should be implemented if necessary.
10. Exercise should be resumed in a stepwise manner after 3 days of rest in asymptomatic COVID-19 patients, after 7 days of complete disappearance of symptoms in COVID-19 patients with mild to moderate symptoms. In patients with severe cardiopulmonary symptoms, exercise should be only considered after cardiac triad test (ECG, echocardiogram, and myocardial enzyme profile): stepwise exercise could be considered after 14 days of complete disappearance of symptoms and normal test, CMR is recommended with abnormal test for possible myocarditis. A repeat cardiac triad test is recommended upon re-emergence or worsening of cardiopulmonary symptoms during return to exercise. Exercises are forbidden for 3–6 months in patients with myocarditis associated with COVID-19.

## AUTHOR CONTRIBUTIONS

JBG, YH, and YWX participated in the consensus design. All authors participated in the performance of the research. YZ, YFZ, JHZ, SZ, and MR participated in previous research collection. YZ, YFZ, DWW, KH, JHZ, SZ, and MR participated in the writing of the paper.

## CONFLICTS OF INTEREST STATEMENT

Junbo Ge is the Editor-in-Chief and Yong Huo is the Associate Editor of *Cardiology Plus*.

## DATA SHARING STATEMENT

Data sharing is not applicable to this article as no datasets were generated or analyzed during the current study.

### Writing Members

Yi Zhang, Yifan Zhao, Daowen Wang, Kai Huang, Jianhui Zhuang, Song Zhao, Rusitanmujiang Maimaitiaili.

### Expert Group Members (in alphabetical order of the surname)

Feng Bai (The Second Hospital of Lanzhou University, Lanzhou 730030, China), Jun Pu (Renji Hospital Affiliated to Shanghai Jiaotong University School of Medicine, Shanghai 200025, China), Wenliang Che (Shanghai Tenth People’s Hospital, Shanghai 200072, China), Jiyan Chen (Guangdong Provincial People’s Hospital, Guangzhou 510000, China), Mao Chen (West China Hospital, Sichuan University, Chengdu 10041, China), Wei Chen (Shanghai Fourth People’s Hospital, Shanghai 200434, China), Xiaoping Chen (Sichuan University West China Hospital, Chengdu 610041, China), Yundai Chen (First Medical Center, PLA General Hospital, Beijing 100038, China), Xianwu Cheng (Yanbian University Affiliated Hospital, Yanji 133099, China), Xiang Cheng (Union Hospital Affiliated to Tongji Medical College, Huazhong University of Science and Technology, Wuhan 430022, China), Hongliang Cong (Tianjin Chest Hospital, Tianjin 300350, China), Cuilian Dai (Cardiovascular Hospital Affiliated to Xiamen University, Xiamen 361004, China), Dali Fan (University of California, Davis Medical Center, Sacramento, CA 95817, USA), Guosheng Fu (Shawyifu Hospital Affiliated to Zhejiang University School of Medicine, Hangzhou 310016, China), Lei Gao (Virginia Mason Franklin Medical Center, Seattle, WA 98101, USA), Chuanyu Gao (Fuwai Huazhong Cardiovascular Hospital, Zhengzhou 451460, China), Wei Gao (Peking University Third Hospital, Beijing 100191, China), Junbo Ge (Zhongshan Hospital Affiliated to Fudan University, Shanghai 200032, China), Ben He (Shanghai Chest Hospital, Shanghai 200030, China), Tao Hu (Xijing Hospital Affiliated to Air Force Medical University, Xi’an 710032, China), Congxin Huang (People’s Hospital of Wuhan University, Wuhan 430060, China), Jing Huang (Second Affiliated Hospital of Chongqing Medical University, Chongqing 400010, China), Yong Huo (First Hospital of Peking University, Beijing 100035, China), Shaobin Jia (General Hospital of Ningxia Medical University, Yinchuan 750003, China), Jun Jiang (Second Affiliated Hospital of Zhejiang University School of Medicine, Hangzhou 310003, China), Zhicheng Jing (Peking Union Hospital, Chinese Academy of Medical Sciences, Beijing 100005, China), Xiangqing Kong (First Affiliated Hospital of Nanjing Medical University, Nanjing 210029, China), Lang Li (First Affiliated Hospital of Guangxi Medical School, Nanning 530021, China), Yan Li (Second Affiliated Hospital of Air Force Medical University, Xi’an 710038, China), Yigang Li (Xinhua Hospital Affiliated to Shanghai Jiao Tong University School of Medicine, Shanghai 200092, China), Zhijuan Li (First Affiliated Hospital of Henan University of Science and Technology, Luoyang 471003, China), Chun Liang (Shanghai Changzheng Hospital, Shanghai 200003, China), Xianhe Lin (First Affiliated Hospital of Anhui Medical University, Hefei 230022, China), Xianxia Liu (Second Affiliated Hospital of Hainan Medical College, Haikou 570216, China), Xuebo Liu (Tongji Hospital Affiliated to Tongji University, Shanghai 200065, China), Chengzhi Lu (Tianjin First Central Hospital, Tianjin 300190, China), Genshan Ma (Zhongda Hospital of Southeast University, Nanjing 210009, China), Yitong Ma (The First Affiliated Hospital of Xinjiang Medical University, Wulumuqi 830011, China), Wei Mao (The First Affiliated Hospital of Zhejiang University of Traditional Chinese Medicine, Hangzhou 310018, China), Xia Mei (Chongqing People’s Hospital, Chongqing 401121, China), Zhongping Ning (Zhoupu Hospital Affiliated to Shanghai Health Medical University, Shanghai 201318, China), Jiafu Ou (Washington University in St. Louis, Saint Louis, MO 63130, USA), Shaderdin Slaj (Hotan District People’s Hospital of Xinjiang, Hetian 848007, China), Chengxing Shen (Sixth People’s Hospital Affiliated to Shanghai Jiaotong University School of Medicine, Shanghai 200233, China), Haiming Shi (Huashan Hospital Affiliated to Fudan University, Shanghai 200040, China), Hong Shi (Chinese Video Journal of Cardiology, Beijing 100052, China), Bei Shi (Affiliated Hospital of Zunyi Medical University, Zunyi 563099, China), Xi Su (Wuhan Asia General Hospital, Wuhan 430056, China), Ningling Sun (Peking University People’s Hospital, Beijing 100036, China), Qizhu Tang (Wuhan University People’s Hospita, Wuhan 430060, China), Fang Wang (Shanghai First People’s Hospital, Shanghai 200940, China), Changqian Wang (Ninth People’s Hospital Affiliated to Shanghai Jiao Tong University School of Medicine, Shanghai 200011, China), Jin Wang (St. Louis Mercy Hospital, Saint Louis, MO 63141, USA), Yanqing Wu (Second Affiliated Hospital of Nanchang University, Nanchang 330008, China), Yongjian Wu (Fuwai Hospital, Chinese Academy of Medical Sciences, Beijing 100037, China), Yunlong Xia (First Affiliated Hospital of Dalian Medical University, Dalian 116011, China), Dingcheng Xiang (General Hospital of the Southern Theater Command, Guangzhou 510016, China), Pingxi Xiao (Shawyifu Hospital Affiliated to Nanjing Medical University, Nanjing 211112, China), Ping Xie (People’s Hospital of Gansu Province, Lanzhou 730099, China), Dingding Xiong (National Children’s Hospital Toledo Branch, Toledo, OH 43608, USA), Yawei Xu (Shanghai Tenth People’s Hospital, Shanghai 200072, China), Jiefu Yang (Beijing Hospital, Beijing 100730, China), Lixia Yang (The 920th Hospital of the PLA Joint Logistics Support Force, Kunming 650032, China), Zaixin Yu (Xiangya Hospital of Central South University, Changsha 410008, China), Zuyi Yuan (The First Affiliated Hospital of Xi’an Jiaotong University, Xi’an 710061, China), Haitao Yuan (Shandong Provincial Hospital, Jinan 250021, China), Guogang Zhang (The Third Xiangya Hospital of Central South University, Changsha 410013, China), Heng Zhang (The First Affiliated Hospital of Bengbu Medical College, Bengbu 233099, China), Jian Zhang (Fuwai Hospital, Chinese Academy of Medical Sciences, Beijing 100037, China), Li Zhang (Xinhua Hospital Affiliated to Shanghai Jiaotong University School of Medicine, Hangzhou 310009, China), Ruiyan Zhang (Ruijin Hospital Affiliated to Shanghai Jiaotong University School of Medicine, Shanghai 200025, China), Shuning Zhang (Zhongshan Hospital Affiliated to Fudan University, Shanghai 200032, China), Shuyang Zhang (Peking Union Hospital, Chinese Academy of Medical Sciences, Beijing 100037, China), Zheng Zhang (The First Hospital of Lanzhou University, Lanzhou 730013, China), Guoan Zhao (The First Affiliated Hospital of Xinxiang Medical College, Xinxiang 453199, China), Xianxian Zhao (Shanghai Changhai Hospital, Shanghai 200433, China), Jingang Zheng (China-Japan Friendship Hospital, Beijing 100029, China), Haoyi Zheng (San Franciscan Heart Center, Roslyn, NY 11576, USA), Daxin Zhou (Zhongshan Hospital Affiliated to Fudan University, Shanghai 200032, China), Shenghua Zhou (Second Xiangya Hospital of Central South University, Changsha 410011, China), Yujie Zhou (Beijing Anzhen Hospital Affiliated to Capital Medical University, Beijing 100029, China).
